# Viewing both sides of the coin for infectious disease diagnosis

**DOI:** 10.1172/JCI169242

**Published:** 2023-04-17

**Authors:** Fiona R. Strouts, Linda B. McAllister, Ephraim L. Tsalik

**Affiliations:** 1 Cepheid, Sunnyvale, California, USA.; 2 Danaher Diagnostics, Washington, DC, USA.; 3 Durham VA Health Care System, Durham, North Carolina, USA.; 4 Duke University School of Medicine, Durham, North Carolina, USA.

## Abstract

Optimal management of lower respiratory tract infection relies on distinguishing infectious from noninfectious etiologies and identifying the microbiologic cause if applicable. This process is complicated by overlapping clinical symptoms and the colonizing lung microbiota. In a recent issue of the *JCI*, Mick, Tsitsiklis, and colleagues apply RNA-Seq to tracheal aspirates from critically ill children and demonstrate how integration of the host response with microbial identification results in a harmonious and accurate diagnostic classifier. Though promising, there are numerous barriers to realizing a combined host and pathogen diagnostic.

## A multipronged approach

Defining the presence of a lower respiratory tract infection (LRTI) in children is challenging due in part to low rates of pathogen detection by conventional microbiologic testing (approximately 20%) (1). Although viruses are identified at high rates in pediatric pneumonia, the clinical importance of these pathogens can be nebulous given high rates of shedding among asymptomatic children and the occurrence of bacterial-viral coinfections (2, 3).

To address these challenges, Mick and colleagues, in a recent issue of the *JCI*, developed a multipronged approach that distinguished pediatric patients with LRTIs from those with alternative diagnoses (including noninfectious illness and nonpulmonary infections), while simultaneously identifying the microbiological cause of the LRTI (4). Other studies combining the host immune response with microbial metagenomic next-generation sequencing (mNGS) have focused on RSV (5), adult LRTI (6), sepsis (7), and tuberculous meningitis (8). In this study, the authors leveraged a previously enrolled cohort of critically ill children aged 31 days to 18 years with acute respiratory failure requiring mechanical ventilation and from whom tracheal aspirate samples were available (4). Of 261 children with confirmed infection, 95% were intubated within two days of admission, timing that is consistent with community-acquired pneumonia. The tracheal aspirate samples were used to generate transcriptomic data, and the resulting sequences were digitally separated into human and nonhuman reads. The authors then integrated three elements to distinguish patients with LRTI from those without LRTI: (a) a 14-mRNA host gene expression classifier; (b) a viral score based on abundance of viral reads and the likelihood of that virus being a respiratory pathogen; and (c) a bacterial score based on the relative abundance of bacterial or fungal reads compared with the nonhuman background. The bacterial score also incorporated microbiome diversity, previously demonstrated to be reduced in the setting of infection (6).

## The host response

Mick and colleagues identified host gene expression changes in a tracheal aspirate that distinguished patients with what they called “Definite” LRTI from those with “No Evidence” of LRTI. Patients with LRTI demonstrated increased expression of genes involved in the immune response to infection and the interferon response and decreased expression of pathways related to protein translation, cilium assembly, and lipid metabolism. Several gene sets were identified (size range, 11–25 genes) that maximized the classification of patients and demonstrated a median area under the receiver operating curve (AUC) of 0.967 by cross validation, with a sensitivity of 92% and specificity of 80% using a defined probability threshold.

The performance of the gene expression classifier to distinguish patients with LRTI from those without infection is indeed encouraging. A substantial advantage of this approach is the small size of the classifier, with as few as 6 genes required, suggesting that it could be deployed on existing quantitative real-time PCR testing platforms that are rapid and easy to use.

Comparing this pediatric host response signature to one developed in an adult cohort (5), there was no overlap in differentially expressed genes. Nevertheless, it is possible the pediatric signature would perform well in the adult cohort or vice versa, because many combinations of differentially expressed genes can substitute for one another in a given classification task (9), though this possibility was not evaluated in Mick et al. (4). Ideally, a universal, age-agnostic signature could be identified for future development. The study also raises the question of whether a host response diagnostic is more sensitive and specific when sampled directly from the site of infection (i.e., tracheal aspirate) as compared with circulating peripheral blood, which is the focus of most host response tests in development.

## Pathogen detection

Microbial mNGS provides a broad screen for potential pathogens that is not available by standard PCR panels or culture. To distinguish potential bacterial and fungal pathogens from commensals, Mick and colleagues applied a rules-based model that ranked high-abundance species relative to the abundance of background species (4, 6). 70% of the samples showed concordance with culture. Of discordant samples, 11% identified a different pathogen, and in the remaining 19%, no pathogen was identified (i.e., false negatives). In 50% of patients with culture-negative LRTI, a potential pathogen was identified by mNGS. This gain in identifiable pathogens is exciting, as it substantially expands the number of cases in which treatment can be tailored to the microbiological etiology. However, it must be tempered by the high rate of false positives: 34% of patients without LRTI had a possible pathogen identified. Perhaps this circumstance is where host response can be particularly impactful, which is precisely what the authors showed. A combined microbial mNGS and host-response classifier reduced the false-positive rate from 34% to 12%. Considering that 84% of patients without LRTI were treated with antibiotics (perhaps unnecessarily), a 12% false-positive rate for infection among patients without LRTI may represent opportunities to limit unnecessary antimicrobial use.

In contrast to bacterial/fungal pathogens, concordance for viral pathogens was 92% among patients with LRTI using a nasopharyngeal swab PCR as the reference. The clinical importance of viral detection among the group without LRTI is unclear, as 16% of individuals in the group without LRTI showed detectable virus. Considering this high concordance between PCR and mNGS, the high cost and complexity of sequencing makes it less appealing than current PCR-based methods if only considering viral pathogen detection.

## Seeing both sides of the coin

Host response biomarkers are emerging as a way to fill several diagnostic gaps. When pathogen detection tests are negative despite clinical evidence of infection, host response can confirm the presence and cause of that infection. Host response also provides context when microorganisms of unclear clinical importance are identified, such as through metagenomic approaches. The study presented by Mick, Tsitsiklis, and colleagues (4) goes beyond these scenarios by revealing how analysis of the host response and metagenomics can be combined synergistically. By simultaneously viewing both sides of the coin, host and pathogen, they generated a harmonious characterization of LRTI status with exceptional accuracy. The combined host and microbial NGS classifier had a cross-validated AUC of 0.986 (4). A similar study by this research team showed an overall accuracy of 100% for a combined host-pathogen classifier in adults (6). While the study by Mick, Tsitsiklis, and colleagues (4) focused on community-acquired LRTI, there is also a clinical need for those with suspected ventilator-associated pneumonia, which was not studied here. Patients with ventilator-associated pneumonia will have both a higher microbial burden in a tracheal aspirate (commensal or colonizing flora) and a higher likelihood of a dysregulated immune response, potentially making both the host and pathogen components of the classifier less reliable.

Questions about the validity and generalizability of this host/pathogen approach can and should be addressed in future research. Answering these questions is necessary but is not sufficient to introduce a new diagnostic paradigm for LRTI or other infectious syndromes.

## A paradigm shift is years in the making

In 2009, Octavio Ramilo and Asunción Mejías described in a Commentary how using host gene profiles to diagnose respiratory infections was a paradigm shift (10). Prophetically, they wrote, “Combining the detection of the pathogen with a comprehensive assessment of the host immune response will provide a broad new understanding of the correlations between specific etiologic agents, the corresponding host response, and the clinical manifestations of the disease.” Fourteen years later, we are still waiting. Studies such as that by Mick, Tsitsiklis, and colleagues (4) and many others clearly show there is potential. Nevertheless, a great deal more is needed to implement this paradigm shift. There are factors that are both intrinsic and extrinsic to the test that must be overcome to realize this potential (Table 1).

Among the test-intrinsic challenges, the most difficult is how to measure the host response in a clinically impactful manner. These measurements are more feasible for protein biomarkers for which immunoassay technologies are well established. Examples of protein host response biomarker profiles include FebriDx (not available in the US) or MeMed BV (licensed to Diasorin and Beckman Coulter), which measure MxA/CRP or IP10/TRAIL/CRP, respectively, to discriminate bacterial and viral infections. While mRNA host response signatures have been well described in the scientific literature and the technologies to measure these mRNA signatures are well established (e.g., quantitative real-time PCR), these tests are technically challenging to develop. They require a high degree of multiplexing, should be simple to perform with rapid turnaround times to inform real-time clinical decisions, and must provide quantitative results with high analytical precision. Only one host mRNA test for infectious diseases has been cleared by the FDA: SeptiCyte RAPID (Immunexpress), measured on the Biocartis Idylla platform, which aids in the discrimination of sepsis from systemic inflammatory response syndrome. Several other companies are making advances but have not yet commercialized tests (e.g., Biomeme, bioMerieux, Cepheid, Inflammatix, and Qvella). The test envisioned by Mick, Tsitsiklis, and colleagues (4) requires sequencing, which is not sufficiently simple, fast, or affordable to justify routine use. Performing mNGS is a complex process that requires skilled personnel, expensive equipment, and a curated database and analysis pipeline. Regardless of the technologies used to measure these signatures, there are no standards for reporting results.

Extrinsic considerations represent the greater challenge, particularly since the field of host-response diagnostics is still emerging. Which endpoints matter to the regulatory authorities, laboratorians, clinicians, patients, and payors? Each stakeholder may have different priorities. Several factors may affect the validation and implementation of these emerging tests (Table 1).

When one considers these intrinsic and extrinsic factors (in addition to many more unspecified), it is no surprise that progress has been slow. As the study by Mick, Tsitsiklis, and colleagues (4) and many others reveal, the opportunity for a comprehensive host and pathogen diagnostic solution is real. Despite a slow start, development is quickening. Hopefully, fourteen years will not have elapsed before someone else marks these words as prophetic.

## Figures and Tables

**Table 1:**
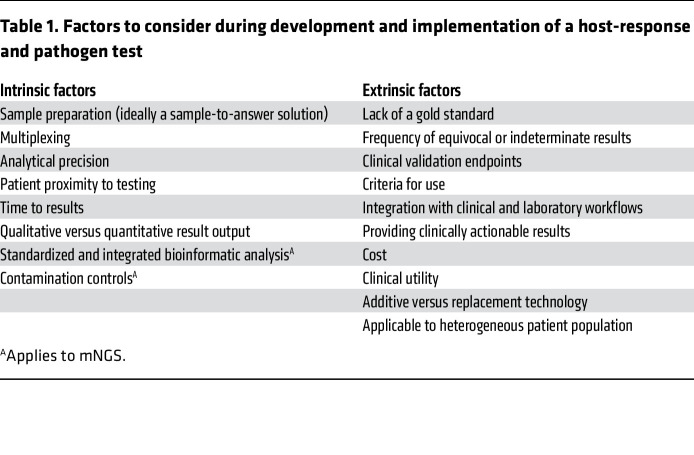
Factors to consider during development and implementation of a host-response and pathogen test

## Version 1 04/17/2023

Electronic publication
